# The Yin and Yang of Innate Immunity in Stroke

**DOI:** 10.1155/2014/807978

**Published:** 2014-04-30

**Authors:** Xiaomeng Xu, Yongjun Jiang

**Affiliations:** Department of Neurology, Jinling Hospital, Nanjing University School of Medicine, Nanjing, Jiangsu 210002, China

## Abstract

Immune system plays an elementary role in the pathophysiological progress of ischemic stroke. It consists of innate and adaptive immune system. Activated within minutes after ischemic onset, innate immunity is responsible for the elimination of necrotic cells and tissue repair, while it is critically involved in the initiation and amplification of poststroke inflammation that amplifies ischemic damage to the brain tissue. Innate immune response requires days to be fully developed, providing a considerable time window for therapeutic intervention, suggesting prospect of novel immunomodulatory therapies against poststroke inflammation-induced brain injury. However, obstacles still exist and a comprehensive understanding of ischemic stroke and innate immune reaction is essential. In this review, we highlighted the current experimental and clinical data depicting the innate immune response following ischemic stroke, mainly focusing on the recognition of damage-associated molecular patterns, activation and recruitment of innate immune cells, and involvement of various cytokines. In addition, clinical trials targeting innate immunity were also documented regardless of the outcome, stressing the requirements for further investigation.

## 1. Introduction

Stroke, among which ischemic stroke accounts for over 87% [1], is the leading cause of morbidity and permanent disability in adults worldwide [2]. It causes severe burdens to the individuals as well as the society, especially in the developing countries like China [3]. However, despite all the intensive researches in the recent decade, the therapeutic strategies of acute ischemic stroke remain limited. Intravenous delivery of recombinant tissue plasminogen activator (rt-PA) is by far the only therapy proved effective in clinical application [4]. Yet this therapy is faced with major drawbacks, the narrow therapeutic time window of 4.5 hours, and the increased risk of intracranial hemorrhage. In the cases delayed beyond the currently approved time window, rt-PA is not warranted since the potential risk would outweigh benefits [5]. Actually, only less than 5% of patients would benefit from the therapy [6]. Therefore, developing alternative therapies is imperative but impossible without a comprehensive understanding of the pathophysiological changes after the ischemic onset.

Ischemic onset is an insult for brain and immune system is firewall for the whole body. Immune system is divided into innate and adaptive systems. The innate immune system is the first line of defense, while immune system and central nervous system (CNS) were traditionally regarded as two distinct entities [7]. The existence of blood-brain barrier and absence of cerebral lymphatic vessels are largely impeding the communication between brain tissue and circulating immune cells and the antigen presenting T cells [8]. However, mounting evidence is challenging this viewpoint, indicating that ischemic stroke is complicated by mutual interplay between CNS and immune system.

Innate immune response plays a dual role in stroke, exerting beneficial as well as deleterious effects on the outcome [9]. Considering the Yin and Yang effects of innate immune system, an overall suppression or activation of innate immunity might not be beneficial, while the true challenge is to selectively inhibit the deleterious effects without compromising the beneficial roles of innate immune response in tissue repair, remodeling, and recovery. It means that we should use immune system after stroke in the right time and right place.

This review mainly focused on latest research data concerning the activation of innate immune response after cerebral ischemia and function of these components.

## 2. Innate Immune System

Immune system monitors and preserves the homeostasis of CNS under normal and pathological conditions. Immune system consists of two mechanisms: the innate and adaptive immunity. The former reacts rapidly after ischemic insult and represents first step of inflammatory cascade [10], while the latter depends on antigen presenting and takes days to be activated. Therefore, innate immunity lays the foundation of the adaptive response and plays the key role in the integrated immune response secondary to cerebral ischemia.

The innate immune system in the brain relies on various immune cells including resident cells such as microglia and endothelia, as well as circulating immune cells from blood such as neutrophils, monocytes/macrophages, and dendritic cells, among which microglia, neutrophils, and monocytes/macrophages are most investigated. Besides the cellular component, cytokines are also involved, mainly including interleukin-1*β* (IL-1*β*), interleukin-6 (IL-6), and tumor necrosis factor-*α* (TNF-*α*). Produced by immune cells, these cytokines function to promote as well as quell inflammation, exerting both deleterious and protective roles.

Immediately after cerebral ischemic onset, dying and dead neurons begin to release the so-called damage-associated molecular patterns (DAMPs). Innate immune system senses the DAMPs via broad-specificity receptors and responds to the cerebral ischemic injuries within minutes, and the response remains predominant throughout the first few hours to induce postischemic inflammatory cascade (Table 1) [11].

Under normal condition, the blood-brain barrier forms a nature obstacle to prevent the entrance of circulating immune cells into the brain. However, under ischemic condition, necrotic tissues would cause local inflammation, leading to the release of inflammatory mediators like cytokines, chemokine, nitric oxide, and reactive oxygen species, which eventually leads to dysfunction of the blood-brain barrier, allowing the translocation of circulating immune cells [1].

## 3. The Role of Innate Immune System in Stroke

### 3.1. Initiation of Innate Immune Response: DAMPs and Pattern Recognition Receptors

Neurons are particularly vulnerable to ischemic insult. Shortly after cerebral vascular accident, local ischemia would lead to the destruction of neurons in the ischemic core and peri-infarct zone, resulting in the release of various DAMPs including high mobility group protein B1 (HMGB1), uric acid, heat shock proteins, S100 proteins, DNA, and RNA, which attract and activate neighboring microglia [8] and thereby trigger the postischemic inflammatory cascade.

In spite of the mounting studies on DAMPs [12–15], it is still controversial which molecule represents the most important mediator that triggers the activation of innate immunity. Among all these DAMPs, high mobility group box 1 (HMGB1) stands out as the most investigated molecule [16]. HMGB1 is a nuclear protein normally localized in cell nuclei under the normal condition. Upon ischemia onset, however, neuronal necrosis causes the protein to translocate into the cytosol and then to passively enter the extracellular compartment. In clinical studies, Schulze and colleagues [12] detected an elevated plasma HMGB1 level in patients with acute ischemic stroke and verified a correlation between HMGB1 level and circulating leukocytes. Release of HMGB1 by necrotic neurons in early stage of cerebral ischemia exhibits proinflammatory activity and amplifies inflammatory damage to brain tissue. Whereas intravenous injection of anti-HMGB1 monoclonal antibody would remarkably ameliorated brain infarction in middle cerebral artery occlusion models [13]. Moreover, via electron microscopic observation, Zhang and colleagues [14] directly demonstrated that HMGB1 release induced rapid and drastic disruption of the BBB, followed by significant cerebral edema, which appeared to be in consistence with their findings by MRI. Interestingly, Hayakawa and colleagues [15, 17] found that, during the stage of stroke recovery, HMGB1 mediated beneficial plasticity and enhanced stem and progenitor cell recruitment, proliferation, and differentiation within damaged brain tissue. This effect, however, occurs in a delayed phase and is beyond the scope of this review.

HMGB1, as well as other DAMPs, is reported to induce downstream biological effects via interactions with pattern recognition receptors, including Toll-like receptors (TLRs), widely expressed on surrounding microglia, perivascular macrophages, and cerebrovascular endothelium [18]. The Toll-like receptor (TLR) pathway plays a pivotal role in the activation and amplification of innate immune response to endogenous tissue damage resulting from cerebral ischemia [19–21]. So far, 10 functional TLRs have been identified in humans as well as 12 in mice. TLR1-TLR9 are conserved in both species, while TLR 10 is not functional in mice because of a retrovirus insertion, and TLR 11, TLR 12, and TLR 13 have been lost from the human genome [22]. Among all the TLRs, TLR 2 and TLR 4 are expressed on the cell surface and detect endogenous ligands [23]. After middle cerebral artery (MCA) occlusion, TLR 2 and TLR 4 are documented to be upregulated and contribute to tissue damage by triggering the expression of inflammatory and apoptotic genes [24]. In fact, TLR 2 and TLR 4 play differential roles in acute cerebral ischemia/reperfusion injury. Hua and colleagues [25] found in genetic modified mice that TLR 4 knockout resulted in reduced infarct size, while TLR 2 knockout led to enlarged infarct size, higher mortality, and decreased neurological function, suggesting that TLR 4 contributed to cerebral ischemia/reperfusion damage, whereas TLR 2 appeared to be neuroprotective in response to cerebral ischemia.

In addition to TLRs, the intracellular NOD-like receptors (NLRs) have also recently been identified as key mediators of inflammatory and immune responses [26]. NLRP 3 contributes to neurovascular damage by regulating the release of NLRP3-mediated proinflammatory mediators, and NLRP3 deficiency ameliorates cerebral ischemic injury in mice after by reducing infarcts and blood-brain barrier damage [27].

### 3.2. Activation of Innate Immune System

#### 3.2.1. Activation of Local Resident Microglia in Central Nervous System

Microglia are the resident macrophages in brain that survey the CNS and eliminate debris via phagocytosis under normal and pathological conditions. In the resting state, microglia exhibit ramified appearance and once activated, these cells alter into an amoeboid morphology. Microglial activation is the initial step in CNS inflammation of numerous causes [9, 28–31]. In ischemic stroke, microglia are activated within minutes of ischemic onset and microglial products are detected as early as 1 hour after stroke [32]. Microglia express pattern recognition receptors including TLRs and NLRs to sense exogenous pathogens and endogenous danger signals [33].

#### 3.2.2. Infiltration of Immune Cells from Peripheral Blood

*Monocytes/Macrophages Accumulation.* Monocytes are resting innate immune cells derived from the blood. Upon activation, these cells would undergo morphological and functional alteration and then be referred to as macrophages. Of note, it has been controversial for years regarding the precise origin of local infiltrating macrophages [34–36], due to the morphological and functional similarity between activated microglia and recruited monocytes/macrophages. Once activated, microglia alter their morphology and gene expression to develop an inflammatory phenotype, making themselves indistinguishable to circulating macrophages [33]. Yet one mostly recent research has settled the debate, proving that local reactive macrophages consist of 2 distinct populations of cells, that is, a majority originates from resident microglia and a small group recruited from circulation [37]. In contrast to the immediate response of microglia, the latter group of cells is recruited no sooner than 2 days after ischemia and remains abundant through day 3 to day 7 [38].

*Neutrophil Infiltration.* Within the acute phase of ischemic stroke (minutes to hours), the injured tissue would release free-radicals and proinflammatory cytokines and chemokine, which would thereby upregulate adhesion molecules on endothelial cells as well as the surface of circulating immune cells, and facilitate the recruitment and migration of leukocytes [38]. Among all the components in the circulating immune system, neutrophils are the first responders [39] that are reported to react to the acute ischemia within 30 minutes and peak in the first 3 days [32]. Via neutrophil CD11b/CD18 and endothelial ICAM-1 interactions, neutrophils adhere to activated vascular endothelium and infiltrate into the injured area, and blocking of the interactions would result in reduced leukocyte accumulation [39, 40].

*Dendritic Cell Increment.* As a link between the innate and adaptive arms of the immune system, dendritic cells (DCs) are key regulators in many forms of immune response [41, 42], but the regulatory role of DCs in inflammation provoked specifically by stroke has not yet been sufficiently investigated [43]. Kostulas and colleagues [44] stood among the first to provide data on DCs in cerebral ischemia and demonstrated ascending numbers of DCs in the ischemic hemispheres in rat models as early as 1 hour after permanent MCA occlusion. Later on, Gelderblom et al. [45] confirmed this finding by analyzing different subclasses of inflammatory cells using flow cytometric analysis and found in surprise that DCs showed one of the largest increases in cell numbers and accounted for a substantial portion among all the infiltrating immune cells with 20-fold increase on day 3 and still 12-fold on day 7. Consistently, a more recent study carried out by Yilmaz and colleagues [43] demonstrated in patients that the numbers of DCs decreased transiently after stroke; furthermore, by analyzing human cerebral specimens with acute ischemic or hemorrhagic stroke, the authors found numerous DCs locating in the infarct area, supporting the hypothesis that the DCs in circulation were most likely to be their recruitment into the infarcted brain. On the other hand, it is also possible that the part of the DCs found in the lesion originates from local cerebral cells such as microglia [44].

### 3.3. Dual Roles of Innate Immune System

#### 3.3.1. Cells

*Microglia.* Activated microglia function analogously to circulating macrophages, with the ability to eliminate necrotic tissue and secrete proinflammatory cytokines including IL-1*β* and TNF-*α* under ischemic condition, which exacerbate brain damage and promote leukocyte infiltration [38]; on the other hand, these cells also exert a neuroprotective potential by releasing anti-inflammatory cytokines like IL-10 and TGF-*β* to quell inflammation and benefit the outcome [38, 46]. In the emerging concept, microglia are assorted into M1 and M2 phenotypes, like macrophages. The M1 phenotype is referred to as the classically activated phenotype and processes deleterious features by secreting proinflammatory cytokines and presenting antigen to T cells, whereas M2 microglia, the alternatively activated phenotype, are involved in the neuroprotection and tissue repair after ischemic injury [33]. Existing data suggests that overall suppression of microglia fails to benefit experimental outcome but, on the contrary, results in larger infarctions and doubling apoptotic neurons after ischemia [47], indicating the significance of microglia in alleviation and recovery of injury.

*Macrophages. *Traditionally, macrophages are viewed as a noxious component that amplifies ischemic injury and exacerbates secondary progression of ischemic lesions. Monocytes/macrophages are recruited via CCL2/CCR2 axis, and deletion of CCR2 or CCL2 results in smaller cerebral infarcts, reduced monocytes/macrophages infiltration, and less proinflammatory mediator production, indicating a deleterious effect of these cells [48]. But the majority of studies demonstrate that macrophages in the injured region, regardless of the exact origin, are polarized into M1 and M2 phenotypes, and the M2 phenotype would show beneficial effects against ischemic damage [29, 49]. Girard and colleagues [50] reported that macrophages that originate from peripheral monocytes might be cytotoxic, independently of their phenotype, while microglia may be protective. On the other hand, Hu and colleagues [49] demonstrated that the majority of microglia/macrophages within the infarct areas experience an M2-to-M1 shift during the stroke progress. Soon after the ischemia, macrophages of the M2 phenotype were present and exerted neuroprotective effects; while being at the later stage of injury, the M2 phenotype gradually transforms into the M1 phenotype and is involved in neuronal damage.

*Neutrophils.* Although elevated neutrophil accumulation is often observed during cerebral ischemia/reperfusion, the exact pathogenesis role of neutrophil infiltration is uncertain, and blocking the postischemia neutrophil recruitment is not necessarily leading to improved outcome [39]. In current concept, neutrophils confer to a functional heterogeneity and polarize into 2 distinct subsets, in which N1 phenotype mediates deleterious effect, while N2 phenotype exhibits neuroprotective effects [51].

*Dendritic Cells*. To date the exact role of DCs was not defined comprehensively, but most studies suggested that DCs increment was associated with worsened outcome [42–45]. In murine models, the numbers of DC in the brain correlated with the size of the brain lesion after pMCAO [44], whereas in patients with transient ischemic attack, acute ischemic stroke, and acute hemorrhagic stroke, the extent of the decrease of DCs significantly correlated with the clinical stage and the radiological size of stroke [43]. Moreover, suppression of DC migration and maturation by granulocyte-colony stimulating factor contributed to attenuation of cerebral inflammation and reduction of infarct size, exhibiting neuroprotective effects in murine models of tMCAO [42].

The mechanism of how DCs lead to poorer outcome remained elusive. Theoretically, DCs presented in the infarcted areas may activate T cells, induce a long-lasting immune response, and therefore lead to further neurological damage [44]. Additionally, the transient decrease of circulating DCs might lead to immunodepression, resulting in poststroke infections to worsen the clinical outcome in stroke patients [43].

#### 3.3.2. Cytokines

Infiltration and activation of innate immune cells result in the production of various cytokines and inflammatory mediators, which either exacerbate or alleviate inflammatory damage to the ischemic brain tissue. Within the first 24 hours of cerebral ischemia in animal models, inflammatory cytokines interleukin-1*β* (IL-1*β*), interleukin-6 (IL-6), and tumor necrosis factor-*α* are upregulated dramatically by up to 40- to 60-fold and are believed to affect the infarct volume and tissue damage. Therefore, these cytokines stand among the most investigated inflammatory mediators [52].

IL-1*β* is one of the neurotoxic cytokines released within 30 minutes after ischemic onset [16]. Activated microglia appear to be the major source, whereas other immune cells may also express IL-1*β* [53]. Noxious effects of IL-1*β* are well documented in numerous studies [1, 53, 54]. It is considered as a neurotoxic mediator that directly induces neuronal death and enhances the expression of cytokines. Furthermore, chronic release of IL-1*β* is associated with increased expression of adhesion molecules and blood-brain barrier permeability, promoting further leukocyte infiltration [55]. In animal experiments, IL-1*α* and IL-1*β* double knockout significantly reduced infarct volume in cerebral ischemic mice models [56]. Additionally, meta-analysis of animal model studies also revealed that IL-1 receptor antagonist markedly reduced infarct volume by 38.2% [57].

Expressed within the first hour after ischemic onset [16], TNF-*α* is also an essential component involved in the early stage of cerebral ischemia [16, 38, 58, 59]. Increased TNF-*α* level in serum was observed after stroke in patients, and the increase correlated infarct volume and severity of neurological impairment [38]. TNF-*α* plays a dual role in brain injury. The neurotoxic effect of TNF-*α* might be attributed to direct induction of neuronal death and indirect promotion of leukocyte infiltration by elevating the expression of adhesion molecules and chemokine. However, in addition to the deleterious roles, TNF-*α* also exerts beneficial effects and mitigates inflammatory injury. TNF receptor knockout was reported to be associated with enlargement of infarct volume [16]. Besides, TNF-*α* pretreatment would result in decreased infarct volume and reduced leukocyte infiltration after permanent middle cerebral artery occlusion in mice [60].

Compared with the former ones, reports on the role of IL-6 in experimental ischemic stroke are relatively fewer [61–65]. And current available evidence argues against a pathogenic role of IL-6 in ischemic stroke. On the contrary, initial studies indicated that IL-6 deficient has no impact on infarct volume in mice models [63]. Whereas studies later on argued that the failure of IL-6 deficient to affect infarct size might be due to hypothermia in the mice models, and with well-controlled hypothermia, IL-6 deficiency would lead to increased infarct volume and neuronal death, suggesting a neuroprotective role of IL-6 [11, 61]. Furthermore, underlying mechanisms of the neuroprotective potential of IL-6 are partially revealed in recent studies, in which IL-6 was demonstrated to participate in angiogenesis [64], and Jung and colleagues [62] verified that IL-6 exerted this ability and protected ischemic tissue probably via STAT3 pathway. Moreover, in order to depict the interactions between various cytokines participating the postcerebral ischemic inflammation, Zeng and colleagues [65] adopted Bayesian network (BN) learning procedure to explore the underlying links among circulating cytokines and discovered that IL-6 modulated TNF-*α* and IL-1*β* mRNA expression directly or indirectly, indicating that IL-6 is a key mediator of the inflammatory cytokine network during the postcerebral ischemia inflammation.

*Attempts and Difficulties in Bench-to-Bedside Translation.* A better knowledge of poststroke inflammatory response may give birth to novel therapeutic strategies against ischemic stroke. Thrombolysis with rt-PA is the only effective treatment to date. However, due to the time window of 4.5 hours and safety concerns, the portion of stroke patients that would benefit from this treatment is less than 5% [6]. On the other hand, immunomodulatory therapies hold a great potential. Based on current knowledge, inflammatory response reacts immediately after ischemic onset while requiring hours to days to fulfill. Therefore, immunomodulatory treatment would have extended therapeutic window. In addition, immunomodulatory therapy would not increase the risk of hemorrhage. Finally, since the inflammation would be particularly exacerbated upon reperfusion, immunomodulation would ameliorate the potential reperfusion-induced exacerbation secondary to medical intervention and recanalization [18].

As is mentioned above, poststroke inflammation is featured by significant leukocyte infiltration, which is facilitated by the upregulated adhesion molecules. Based on this theory, clinical trials are conducted to explore whether the suppression of leukocyte infiltration by blocking ICAM-1 with monoclonal antibody enlimomab benefits clinical outcome in acute ischemic stroke patients. Disappointingly, the clinical trial ended up with negative results, suggesting an even worsened outcome upon enlimomab treatment [66]. In this study, 625 patients with ischemic stroke were enrolled, of whom 317 were randomized to receive enlimomab within 6 hours after stroke onset. Patients were not enrolled if they had received rt-PA. The treatment lasted over 5 days. However, when evaluated at day 90, patients that received enlimomab exhibited significantly worse Modified Rankin Scale score and higher mortality. Additionally, patients in enlimomab group experienced more adverse events, primarily infections and fever, than the placebo group. The negative effect may be interpreted by the murine source of enlimomab and the murine antibody might activate neutrophils through complement-dependent mechanisms and therefore amplify the inflammation and damage [1].

Likewise, UK-279, 276, a recombinant glycoprotein that selectively binds to the CD11b integrin to reduce neutrophil infiltration and infarct size in murine models, failed to exhibit any benefit in patients. The study was a multicenter, double-blind, randomized, placebocontrolled clinical trial to evaluate the efficacy of UK-279,276 in acute ischemic stroke. 966 patients were enrolled, among whom 887 had ischemic stroke and 204 were cotreated with rt-PA. Unfortunately, the trial was stopped early for futility in both subgroups receiving UK-279,276 no matter with concomitant rt-PA prescription or not [67].

In addition to the above-mentioned immune cells and cytokines that complicate the immune response to stroke, free-radicals also complicate the pathophysiological progress [68, 69]. Therefore, free-radical trapping agents like NXY-059 are theoretically neuroprotective, and this hypothesis was confirmed in animal models [70]. Furthermore, SAINT I study [70] found that NXY-059 significantly reduced disability rate in patients receiving this agent and markedly lowered hemorrhagic risk in those receiving rt-PA concomitantly. Nonetheless, the subsequent SAINT II study [71] overturned both of these optimistic findings, stating that NXY-059 was ineffective for the treatment of acute ischemic stroke and had no effect on the hemorrhagic risk of rt-PA. Since SAINT II study presented larger sample size (3306 versus 1699), it is reasonable to consider the results from SAINT II study to be more reliable and the data from SAINT I may be false positive. However, even though no evidence was found of an interaction between rt-PA use and the effect of NXY-059 in either trial, we cannot completely rule out the possibility that the disparity between the two trials may derive from the higher frequency of rt-PA prescription use in SAINT II study (44% versus 29%) in which maximal improvement may be achieved already by rt-PA, in spite of NXY-059 [71].

There are several promising findings as well. In another trial, investigators adopted IL-1 receptor antagonist (IL-1Ra) in attempt to block cytokine cascade. This randomized phase II study [72] recruited 34 patients, among whom half were randomized to receive IL-1Ra and the others received placebos. None of the patients received rt-PA. Upon 3-month evaluation, patients that received IL-1Ra exhibited lower levels of inflammatory markers and better clinical outcomes. No adverse events were observed in both groups. This study indicated that IL-1Ra was safe and well tolerated among acute stroke patients and that IL-1Ra held a great potential to be a novel therapy, whereas the efficacy required further investigation.

The trial and errors in the attempt to find novel therapeutic strategies targeting poststroke inflammation have revealed several obstacles before successful clinical translation were put forward by Macrez and colleagues [1]. Firstly, it is still uncertain whether animal models of stroke can recapitulate human pathology and predict success in clinical trials needs. Secondly, safety, tolerance, and potential adverse events associated with therapeutic immunomodulation are of relevant concern. Finally, our knowledge of immune system and stroke is still limited. The interactions between stroke and immunity are elusive, and the role of inflammation in ischemic injury is complicated and sometimes conflicting. Therefore, safe and successful bench-to-bedside translation calls for a more comprehensive understanding of immune response after ischemic stroke before it could benefit stroke patients substantially.

## 4. Conclusions

Innate immune system, triggered immediately and kept for a while after ischemic stroke onset, protects and hurts brain by activation of endogenous and exogenous immune cells and production of cytokines. Immunomodulatory therapies targeting the poststroke inflammation are promising with great obstacles, and a comprehensive understanding of innate immune response to cerebral ischemic attack calls for further investigation.

## Figures and Tables

**Table 1:** Activation and function of innate immune cells.

	Activation	Function
Microglia	Within minutes	M1 phenotype promotes inflammation by secreting cytokines like IL-1*β*, TNF-*α* and presenting antigens to T cell; M2 phenotype quells inflammation by secreting cytokines like IL-10, TGF-*β*, and tissue repair [38, 46].

Macrophages	2 days	View point 1: M1 phenotype promotes inflammation and M2 phenotype quells inflammation (similar with microglia) [29, 49]. View point 2: macrophages originating from peripheral monocytes are cytotoxic, while those from microglia are protective [50].

Neutrophils	30 minutes	N1 phenotype exacerbates the damage, whereas N2 is protective [51].

Dendritic cells	1 hour	Generally deleterious, suggested by researches to date [42–45].
